# Supplementary material for: Serum Amino Acid Profiles in Dogs with a Congenital Portosystemic Shunt
Source: Metabolites. 2025 Apr 9;15(4):258. doi: 10.3390/metabo15040258 (PMC12029160; doi:10.3390/metabo15040258)
Supplement: Supplementary file 1 [file metabolites-15-00258-s001.zip › metabolites-3551755_Supplementary.docx]

Supplementary Tables & Figures

**Table S1.** Median [range] concentrations of select serum biochemistry analytes by disease group. Dogs with different congenital portosystemic shunt morphologies are compared by Mann-Whittney tests. Values from healthy control dogs (collected on a different chemistry analyzer) are provided for reference in gray.

| **Analyte** | **HC** | **eCPSS** | **iCPSS** | ***P*-value** |
| --- | --- | --- | --- | --- |
| ALT (U/L) | 38 [9–61] | 110 [38–1252] | 217 [29–847] | 0.6920 |
| Albumin (g/dL) | 3.3 [2.8–3.9] | 2.5 [1.5–3.3] | 2.3 [1.2–2.8] | 0.1412 |
| BUN (mg/dL) | 16 [9–26] | 7 [3–21] | 5 [3–11] | 0.0688 |
| Cholesterol (mg/dL) | 194 [135–255] | 116 [47–251] | 116 [50–239] | 0.7515 |
| Glucose (mg/dL) | 94 [76–101] | 96 [52–150] | 94 [68–191] | 0.6554 |

Abbreviations: HC, healthy controls; eCPSS, extrahepatic congenital portosystemic shunt; iCPSS, intrahepatic congenital portosystemic shunt.

**Table S2.** Summary of medical management before surgical intervention for dogs with an extrahepatic congenital portosystemic shunt (eCPSS) or an intrahepatic congenital portosystemic shunt (iCPSS). Duration of treatment (in days) is reported as medians [ranges].

|  | | **All CPSS dogs** | **eCPSS dogs** | **iCPSS dogs** |
| --- | --- | --- | --- | --- |
| Total number of dogs: | | 60 | 50 | 10 |
|  | |  |  |  |
| Diet pre- surgery: | Hydrolyzed | 38 | 32 | 6 |
|  | Liver | 16 | 13 | 3 |
|  | Urinary | 1 | 1 | 0 |
|  | Non-prescription maintenance | 2 | 2 | 0 |
|  | Unknown | 3 | 2 | 1 |
| Duration of diet (days): | | 31 [5–1095] | 31 [5–1095] | 42 [26–112] |
|  | |  |  |  |
| Antibiotic(s) used  pre-surgery: | Amoxicillin | 15 | 12 | 3 |
|  | Co-amoxiclav | 35 | 28 | 7 |
|  | Metronidazole | 7 | 6 | 1 |
|  | Doxycycline | 1 | 1 | 0 |
|  | None | 6 | 6 | 0 |
| Duration of antibiotic use (days): | | 33 [5–1209] | 31 [5–1209] | 73.5 [27–1209] |
|  | |  |  |  |
| Lactulose used  pre-surgery: | Yes | 59 | 49 | 10 |
|  | No | 1 | 1 | 0 |
| Duration of lactulose use (days): | | 33 [5–1209] | 31 [5–1209] | 51 [26–112] |
|  | |  |  |  |
| Clinical signs following medical management: | Resolved | 19 | 15 | 4 |
|  | Improved | 31 | 28 | 3 |
|  | Unchanged | 10 | 7 | 3 |
|  | |  |  |  |
| Grade of hepatic encephalopathy (HE) at time of surgery: | 1 – Not present | 46 | 37 | 9 |
|  | 2 – Mild | 9 | 9 | 0 |
|  | 3 – Moderate | 1 | 1 | 0 |
|  | 4 – Severe | 2 | 2 | 0 |
|  | Not available | 2 | 1 | 1 |

Examples of prescription veterinary diets included the following: “hydrolyzed” – Purina HA, Royal Canin HP; “liver” – Hills l/d, Royal Canin hepatic; “urinary” – Hills u/c.

**Table S3.** Concentrations (µmol/L) of serum amino acids (AA), nitrogenous compounds, and calculated amino acids metrics of healthy control (HC) dogs and dogs with an extrahepatic congenital portosystemic shunt (eCPSS) or an intrahepatic congenital portosystemic shunt (iCPSS). Data presented as medians [ranges]. Groups were compared using Kruskal-Wallis tests followed by Dunn’s multiple comparisons. Comparisons significantly different after Benjamini-Hochberg procedure are shaded in gray. Medians not sharing a common superscript are significantly different (at *P* < 0.05).

| **Parameter** | **HC** | **eCPSS** | **iCPSS** | **Corrected *P*-value** |
| --- | --- | --- | --- | --- |
| Ala | 362.3 [214.3–633.5] | 353.3 [161.9–873.5] | 335.6 [136.9–574.6] | 0.7274 |
| Amm | 42.6^a^ [15.0–91.1] | 167.8^b^ [41.7–1974.0] | 151.6^b^ [94.5–261.9] | <0.0001 |
| Arg | 121.5 [67.4–190.3] | 104.8 [0.0–205.8] | 150.1 [77.2–225.4] | 0.3580 |
| Asn | 49.6^a^ [21.4–99.7] | 73.9^b^ [22.2–190.8] | 117.2^b^ [52.9–294.7] | 0.0002 |
| Citr | 29.4 [7.4–89.8] | 39.4 [0.0–212.4] | 51.8 [8.8–148.9] | 0.1881 |
| Gln | 802.0 [582.7–1105.0] | 803.8 [310.5–1620.0] | 868.3 [410.3–1630.0] | 0.9893 |
| Glu | 9.1^a^ [0.0–26.3] | 33.0^b^ [4.0–113.0] | 28.9^b^ [12.7–61.1] | <0.0001 |
| Gly | 223.3 [140.1–403.7] | 206.5 [89.4–361.6] | 214.6 [123.0–384.7] | 0.1783 |
| His | 58.2^a^ [40.8–79.0] | 79.0^b^ [31.8–134.8] | 83.6^b^ [47.5–122.5] | 0.0002 |
| Ile | 49.9^a^ [10.0–72.6] | 32.6^b^ [10.0–93.6] | 10.0^b^ [10.0–57.6] | 0.0022 |
| Leu | 115.9^a^ [75.5–182.0] | 68.0^b^ [31.3–210.1] | 50.3^b^ [10.0–137.1] | <0.0001 |
| Lys | 136.1 [46.3–243.3] | 144.2 [71.1–325.9] | 123.3 [51.8–231.5] | 0.4752 |
| Met | 48.7 [28.3–77.4] | 43.8 [2.3–201.3] | 52.5 [25.3–384.1] | 0.2487 |
| Phe | 39.5^a^ [22.6–64.8] | 95.9^b^ [31.9–212.6] | 113.2^b^ [57.1–171.0] | <0.0001 |
| Pro | 110.5 [53.6–240.1] | 140.1 [64.0–238.7] | 162.6 [70.1–276.5] | 0.1783 |
| Ser | 101.1^a^ [45.0–149.3] | 147.0^b^ [71.2–394.4] | 157.3^b^ [98.6–223.2] | 0.0003 |
| Taur | 196.8 [97.1–275.9] | 207.7 [9.8–475.5] | 187.1 [55.6–361.0] | 0.4230 |
| Thr | 174.0^a^ [40.6–285.8] | 63.3^b^ [0.0–298.8] | 82.0^b^ [0.0–176.9] | <0.0001 |
| Trp | 28.2 [5.0–101.0] | 14.7 [5.0–123.7] | 34.5 [5.0–73.6] | 0.0958 |
| Tyr | 23.0^a^ [0.6–64.9] | 71.5^b^ [16.8–377.9] | 74.7^b^ [40.0–221.9] | <0.0001 |
| Urea | 3176^a^ [1512–6385] | 1681^b^ [786–5629] | 1455^b^ [750–3491] | 0.0010 |
| Val | 161.0^a^ [104.2–231.9] | 97.7^b^ [52.6–262.9] | 68.8^b^ [41.4–143.8] | <0.0001 |
| BCAA | 330.4^a^ [221.6–458.7] | 199.9^b^ [93.9–565.1] | 137.9^b^ [63.9–338.5] | <0.0001 |
| AAA | 64.1^a^ [23.1–129.7] | 172.0^b^ [48.8–473.9] | 184.8^b^ [97.2–393.0] | <0.0001 |
| Fischer | 5.3^a^ [2.6–9.6] | 1.1^b^ [0.3–5.8] | 0.6^b^ [0.2–2.5] | <0.0001 |
| BTR | 14.3^a^ [5.3–24.6] | 2.9^b^ [0.4–19.2] | 1.5^b^ [0.4–6.1] | <0.0001 |

Abbreviations: Ala, alanine; Amm, ammonia; Arg, arginine; Asn, asparagine; Citr, citrulline; Gln, glutamine; Glu, glutamic acid; Gly, glycine; His, histidine; Ile, isoleucine; Leu, leucine; Lys, lysine; Met, methionine; Phe, phenylalanine; Pro, proline; Ser, serine; Taur, taurine; Thr, threonine; Trp, tryptophan; Tyr, tyrosine; Val, valine; BCAA, total branched-chain amino acids (Ile + Leu + Val); AAA, total aromatic amino acids (Phe +Tyr); Fischer, Fischer’s ratio (BCAA/AAA); BTR, BCAA-to-tyrosine ratio.

**Table S4.** Concentrations (µmol/L) of serum amino acids (AA), nitrogenous compounds, and calculated amino acids metrics in 15 CPSS dogs at time of surgery (baseline) and at follow-up appointment. Data presented as medians [ranges]. Timepoints were compared using Wilcoxon matched-pairs signed rank tests. Comparisons significantly different after Benjamini-Hochberg procedure are shaded in gray.

| **Parameter** | **Surgery** | **Follow-up** | **Corrected *P*-value** |
| --- | --- | --- | --- |
| Ala | 272.8 [136.9–545.1] | 364.0 [182.8–801.9] | 0.2089 |
| Amm | 169.4 [41.7–1,974.0] | 80.0 [38.1–616.4] | 0.0181 |
| Arg | 101.1 [17.5–205.8] | 127.0 [3.5–247.1] | 0.1987 |
| Asn | 72.6 [37.4–135.7] | 78.4 [30.5–147.7] | 0.9780 |
| Citr | 43.7 [0.0–212.4] | 47.0 [6.9–129.9] | 0.9715 |
| Gln | 775.2 [540.1–1,426.0] | 846.4 [552.0–1,187.0] | 0.7673 |
| Glu | 29.1 [4.0–84.9] | 25.5 [11.4–47.0] | 0.5764 |
| Gly | 199.0 [89.4–348.6] | 208.9 [124.0–344.0] | 0.7085 |
| His | 75.5 [36.2–116.0] | 66.2 [43.1–106.3] | 0.4235 |
| Ile | 21.9 [10.0–50.3] | 38.9 [10.0–81.3] | 0.0215 |
| Leu | 54.8 [31.3–105.3] | 89.4 [37.1–201.5] | 0.0057 |
| Lys | 117.4 [79.5–207.7] | 176.5 [114.0–264.3] | 0.0044 |
| Met | 44.1 [23.2–201.3] | 39.1 [15.1–97.7] | 0.6494 |
| Phe | 108.2 [31.9–212.6] | 52.0 [26.7–156.3] | 0.0181 |
| Pro | 134.8 [64.0–173.4] | 123.3 [46.5–234.3] | 0.6494 |
| Ser | 142.8 [71.2–312.0] | 135.6 [79.2–185.9] | 0.3377 |
| Taur | 174.8 [98.7–412.5] | 208.6 [82.0–378.4] | 0.5625 |
| Thr | 44.0 [0.5–120.1] | 109.3 [0.0–300.9] | 0.0181 |
| Trp | 20.6 [5.0–77.4] | 19.6 [5.0–114.1] | 0.9715 |
| Tyr | 64.4 [16.8–148.6] | 28.4 [10.7–89.0] | 0.0155 |
| Urea | 1291 [862–3607] | 2850 [913–6599] | 0.0044 |
| Val | 75.9 [52.3–137.6] | 131.6 [57.8–272.3] | 0.0044 |
| BCAA | 147.6 [93.9–293.2] | 260.6 [104.9–555.1] | 0.0057 |
| AAA | 158.6 [48.8–361.2] | 74.6 [40.4–218.0] | 0.0139 |
| Fischer | 0.9 [0.5–4.9] | 3.5 [0.7–7.4] | 0.0044 |
| BTR | 2.2 [1.2–15.8] | 11.6 [1.2–29.3] | 0.0044 |

Abbreviations: Ala, alanine; Amm, ammonia; Arg, arginine; Asn, asparagine; Citr, citrulline; Gln, glutamine; Glu, glutamic acid; Gly, glycine; His, histidine; Ile, isoleucine; Leu, leucine; Lys, lysine; Met, methionine; Phe, phenylalanine; Pro, proline; Ser, serine; Taur, taurine; Thr, threonine; Trp, tryptophan; Tyr, tyrosine; Val, valine; BCAA, total branched-chain amino acids (Ile + Leu + Val); AAA, total aromatic amino acids (Phe +Tyr); Fischer, Fischer’s ratio (BCAA/AAA); BTR, BCAA-to-tyrosine ratio. **Table S5.** Spearman’s r (r_s_) and *P*-values for correlations between serum amino acid concentrations and select biochemistry analytes in dogs with congenital portosystemic shunts. Significant correlations are shaded in gray.

|  | **ALT** | | **Albumin** | | **Ammonia** | | **Cholesterol** | | **Glucose** | |
| --- | --- | --- | --- | --- | --- | --- | --- | --- | --- | --- |
| **Amino Acid** | *r_s_* | ***P-value*** | *r_s_* | ***P-value*** | *r_s_* | ***P-value*** | *r_s_* | ***P-value*** | *r_s_* | ***P-value*** |
| Ala | -0.25 | 0.0714 | 0.04 | 0.7507 | 0.08 | 0.5442 | 0.44 | 0.0012 | -0.09 | 0.5992 |
| Amm | 0.31 | 0.0227 | -0.23 | 0.0903 | 1.00 | <0.0001 | -0.52 | <0.0001 | -0.11 | 0.5131 |
| Arg | -0.25 | 0.0706 | -0.23 | 0.0938 | 0.00 | 0.9951 | 0.08 | 0.5874 | 0.05 | 0.7858 |
| Asn | -0.05 | 0.6963 | -0.23 | 0.0828 | 0.04 | 0.7692 | 0.05 | 0.7071 | 0.00 | 0.9822 |
| Citr | -0.34 | 0.0114 | -0.31 | 0.0207 | -0.04 | 0.7621 | 0.04 | 0.7730 | -0.11 | 0.5113 |
| Gln | 0.12 | 0.3710 | 0.03 | 0.8077 | -0.05 | 0.7012 | 0.22 | 0.1284 | -0.04 | 0.8067 |
| Glu | 0.06 | 0.6865 | -0.02 | 0.8676 | 0.44 | 0.0004 | -0.01 | 0.9227 | 0.02 | 0.9151 |
| Gly | -0.15 | 0.2617 | -0.26 | 0.0567 | 0.18 | 0.1739 | 0.20 | 0.1731 | -0.20 | 0.2443 |
| His | 0.11 | 0.4053 | -0.04 | 0.7549 | 0.16 | 0.2143 | 0.05 | 0.7096 | -0.02 | 0.8941 |
| Ile | 0.05 | 0.7181 | 0.12 | 0.3972 | 0.08 | 0.5610 | 0.20 | 0.1683 | -0.10 | 0.5587 |
| Leu | 0.00 | 0.9885 | 0.15 | 0.2749 | 0.08 | 0.5289 | 0.28 | 0.0526 | -0.01 | 0.9589 |
| Lys | -0.18 | 0.1788 | -0.13 | 0.3376 | 0.15 | 0.2467 | 0.11 | 0.4514 | 0.01 | 0.9500 |
| Met | -0.21 | 0.1271 | -0.06 | 0.6420 | -0.17 | 0.1977 | 0.23 | 0.1075 | 0.23 | 0.1662 |
| Phe | 0.20 | 0.1418 | -0.33 | 0.0134 | 0.19 | 0.1449 | -0.25 | 0.0768 | -0.01 | 0.9439 |
| Pro | -0.36 | 0.0076 | -0.30 | 0.0237 | -0.14 | 0.2805 | 0.28 | 0.0497 | -0.04 | 0.8122 |
| Ser | 0.14 | 0.2966 | -0.16 | 0.2498 | 0.06 | 0.6603 | 0.13 | 0.3640 | 0.07 | 0.6781 |
| Taur | 0.00 | 0.9741 | -0.14 | 0.2901 | 0.33 | 0.0102 | -0.11 | 0.4339 | 0.20 | 0.2243 |
| Thr | -0.23 | 0.0970 | -0.15 | 0.2808 | -0.10 | 0.4453 | 0.37 | 0.0077 | -0.25 | 0.1338 |
| Trp | 0.31 | 0.0225 | 0.02 | 0.8764 | 0.07 | 0.5979 | -0.12 | 0.4004 | 0.09 | 0.6141 |
| Tyr | 0.11 | 0.4145 | -0.26 | 0.0501 | 0.10 | 0.4606 | -0.17 | 0.2261 | -0.18 | 0.2883 |
| Urea | -0.18 | 0.1862 | 0.03 | 0.8532 | -0.05 | 0.6928 | 0.07 | 0.6355 | -0.29 | 0.0765 |
| Val | 0.03 | 0.8390 | 0.17 | 0.1977 | 0.01 | 0.9181 | 0.26 | 0.0654 | -0.07 | 0.6833 |
| BCAA | 0.02 | 0.8828 | 0.15 | 0.2806 | 0.06 | 0.6570 | 0.25 | 0.0831 | -0.05 | 0.7564 |
| AAA | 0.19 | 0.1737 | -0.28 | 0.0366 | 0.17 | 0.2039 | -0.24 | 0.0940 | -0.14 | 0.4156 |
| Fischer | -0.18 | 0.1865 | 0.21 | 0.1247 | -0.08 | 0.5416 | 0.34 | 0.0171 | 0.05 | 0.7662 |
| BTR | -0.15 | 0.2620 | 0.20 | 0.1373 | -0.03 | 0.8098 | 0.30 | 0.0374 | 0.08 | 0.6552 |

Abbreviations: Ala, alanine; Amm, ammonia; Arg, arginine; Asn, asparagine; Citr, citrulline; Gln, glutamine; Glu, glutamic acid; Gly, glycine; His, histidine; Ile, isoleucine; Leu, leucine; Lys, lysine; Met, methionine; Phe, phenylalanine; Pro, proline; Ser, serine; Taur, taurine; Thr, threonine; Trp, tryptophan; Tyr, tyrosine; Val, valine; BCAA, total branched-chain amino acids (Ile + Leu + Val); AAA, total aromatic amino acids (Phe +Tyr); Fischer, Fischer’s ratio (BCAA/AAA); BTR, BCAA-to-tyrosine ratio.

|  |   **B** |
| --- | --- |

**Figure S1.** Representative AA metric (Fischer’s ratio) demonstrating typical changes observed in: (**A**) follow-up dogs who were seen more than 3 months after surgery (orange circles, n = 5) versus those seen less than 3 months after surgery (black circles, n = 10); and (**B**) follow-up dogs who would go on to receive additional surgical intervention (blue circles, n = 6) versus those who did not (black circles, n = 9). Concentrations of healthy control dogs (HC, shaded in gray) are provided as reference. Median for each group is indicated by red line.

**A**
